# Primary extraskeletal Ewing's sarcoma of the maxillary sinus^☆^

**DOI:** 10.1016/j.bjorl.2016.03.014

**Published:** 2016-04-27

**Authors:** Neslihan Yaprak, Havva Serap Toru, Irem Hicran Ozbudak, Alper Tunga Derin

**Affiliations:** a Akdeniz University, School of Medicine, Department of Otorhinolaryngology, Antalya, Turkey; b Akdeniz University, School of Medicine, Department of Pathology, Antalya, Turkey

## Introduction

Ewing's sarcoma was identified by James Ewing in 1921 as a perivascular endothelial tumor.^1^ Since the 1980s, it has been believed that this tumor originates from mesenchymal, myeloid, or primitive multipotential cells.^2^ Although it mostly originates from bones of lower extremities, it can also arise from soft tissues such as paravertebral region. These small round cell tumors have been termed Ewing's Sarcoma (ES)/Peripheral Primitive Neuroectodermal Tumor (PNET) and include ES of the bone, extraskeletal ES, PNET and Askin's tumor.^3^

Extraskeletal ES rarely occurs in the head and neck localization, account for only 1–4% of all ES, and usually prefers mandibula.^4^ However, there are few reports in the literature of ES arising from maxillary sinuses.^4^, ^5^ The gender distribution is equal and nearly half of the patients in age between 10 and 20 years old, while 70% are under the age of 20.^6^ The patients usually presented with painless swelling and symptoms such as anemia, leukocytosis, weight loss, nasal congestion (based on the location), vision loss, and headache.^4^ Herein, we describe an 11 year-old female patient diagnosed as ES in the maxillary sinus. The characteristics of the tumor and the clinical approach are reviewed with the literature.

## Case report

**Figure 1:**
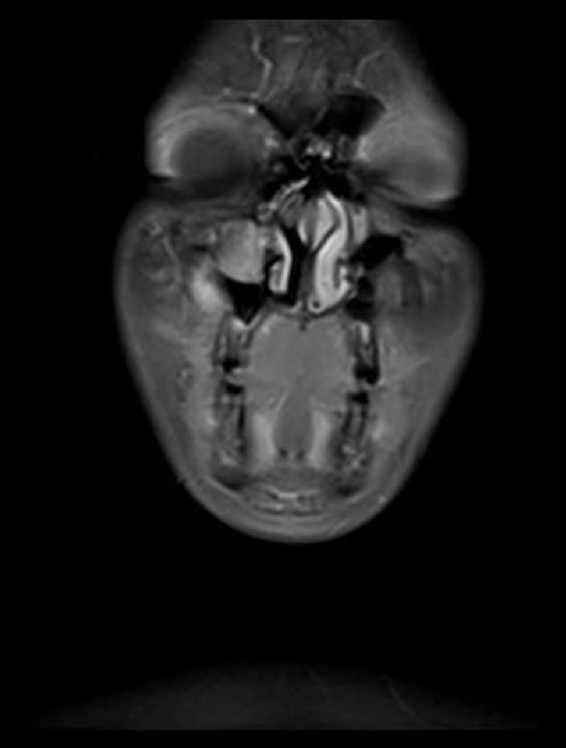
A coronal MRI of the sinuses revealed a mass in the right maxillary sinus.

**Figure 2:**
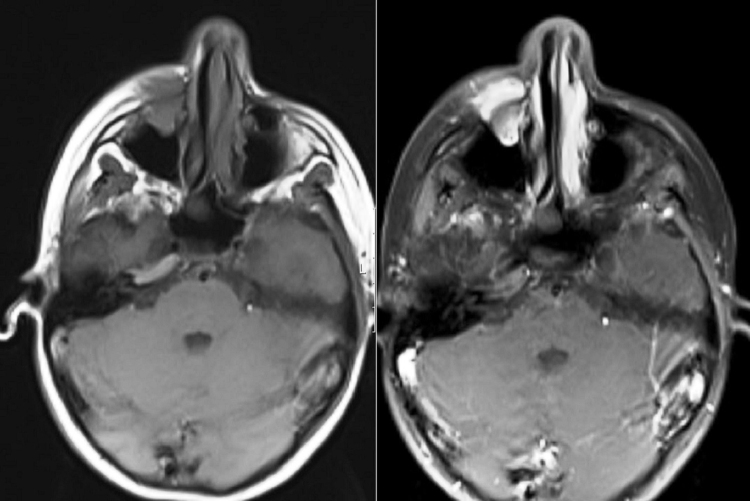
A axial MRI of the sinuses revealed a mass in the right maxillary sinus.

An 11 year-old female patient presented to our clinic with the complaint of swelling on her right cheek. Her history revealed that the swelling had emerged 2 weeks prior and had been growing increasingly since then. In the physical examination, there were no other significant findings except an immobile, hard on palpation 2 × 2 cm mass lesion on the right cheek. Laboratory tests showed no abnormalities. Magnetic Resonance Imaging (MRI) revealed a mass lesion centered in the right maxillary sinus extending to the anterior wall and soft tissue (Figure 1, Figure 2). No metastasis was identified.

On suspicion of malignancy, the patient underwent open surgery by approaching through sublabial incision and subsequently biopsy specimen was submitted to pathology.

**Figure 3:**
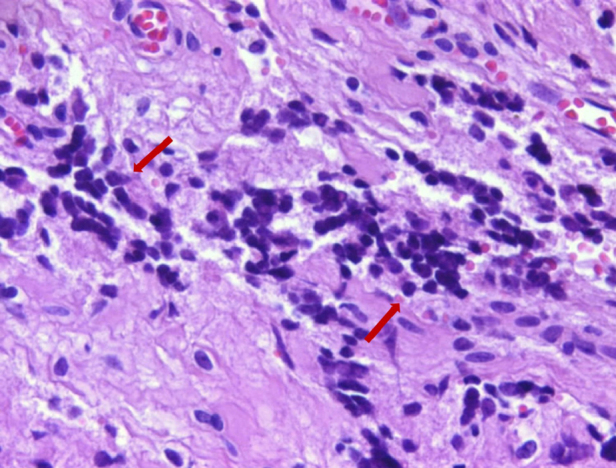
Monoton tumor cells infiltrating stroma. The cells have round nuclei with small and inconspicuous nucleoli (arrows) (hemotoxylin & eosin, ×400).

**Figure 4:**
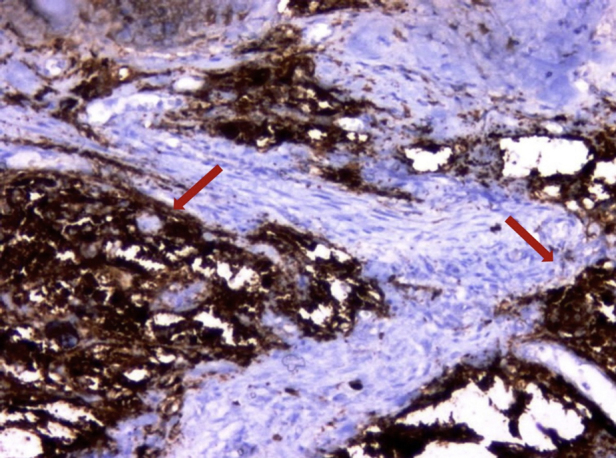
Tumor cells are forming solid areas and expressing synaptophysin immunohistochemically (arrows) (×200). Corrected figure legends and arrows were added to the microphotographs.

In the histopathological examination, the tumor showed closely packed uniform small round cells with high nuclear/cytoplasmic ratios and finely dispersed chromatin, without prominent nucleoli. Mitotic figures were numerous. Areas of necrosis were present. Immunohistochemically, the tumor cells were strongly positive with CD99 and vimentin as well. Occasional clusters had some cells positive for synaptophysin and CD56. The tumor cells were negative with CD3, CD20, LCA, PanCK, myogenin, myoD1 and desmin. Ki-67 proliferative index was found between 30% and 50% in some areas. Subsequently, ES appeared as the most probable diagnosis. Therefore the patient material submitted to the molecular pathology for the evaluation of classical ESWR1-FLI1 rearrangement. The results of Fluorescence In Situ Hybridization (FISH) for ESWR1-FLI1 rearrangement confirmed the diagnosis ES/PNET (Figure 3, Figure 4).

**Figure 5:**
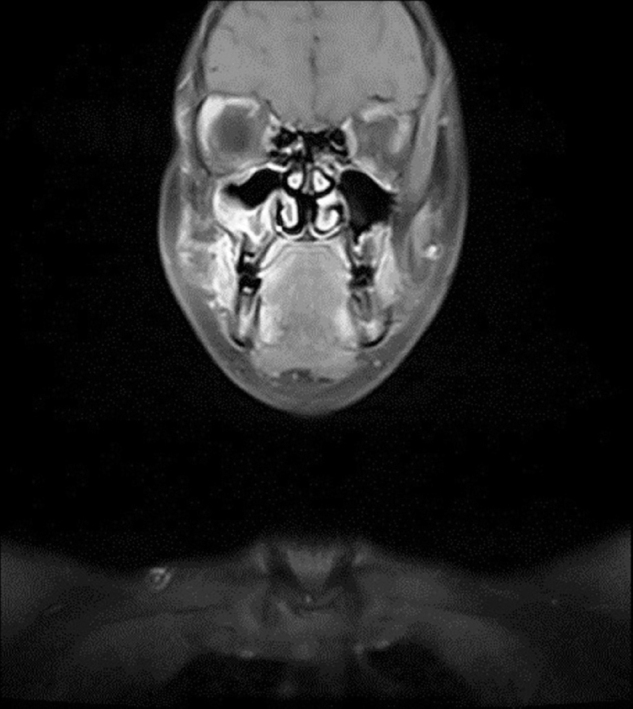
A coronal MRI of the sinuses; mucosal thickening appears in the right maxillary sinus, after treatment.

The patient was treated by chemotherapy. Patient was treated with Ewing's sarcoma VDC/IE (V, vincristine 1.5 mg/m^2^; D, doxorobucin 75 mg/m^2^; C, cyclophosphamide 1.2 g/m^2^; I, Ifosphamide 1.8 g/m^2^; E, etoposide 100 mg/m^2^) Pediatric Oncology Group protocol which were administered 17 cycles over 48 weeks. During the follow-up, after 4 months, local recurrence was detected. The patient underwent open surgery with the same procedure approaching through sublabial incision. The resection was extended to reach safely negative tumor margins. Radiotherapy was added to treatment procedure following the surgery. The metastasis investigation was carried out by Positron Emission Tomography (PET) scan and MRI every 6 months. Also the patient was followed by routine clinical examination every 3 months. There was no finding of recurrence or metastasis at the 13th month of follow-up (Fig. 5).

## Discussion

Extraskeletal ES is a rare tumor in head and neck region. The differential diagnosis includes several entities occurring at the bones and soft tissues of sinonasal tract such as malignant melanoma, rhabdomyosarcoma, sinonasal undifferentiated carcinoma, lymphoma and olfactory neuroblastoma. In our case, considering the patient's age extraskeletal ES, rhabdomyosarcoma and lymphoma were included in the differential diagnosis. The possibility of rhabdomyosarcoma and lymphoma was excluded and the diagnosis of extraskeletal ES was confirmed with immunohistochemical stainings and FISH for ESWR1-FLI1 rearrangement.^7^

A majority of ES have t(11;22)(q24;q12) which corresponds to a fusion between the EWS gene (22q12) and the FLI1 gene (11q24) which is a transcription factor.

Moreover, four different translocations are observed. Those are EWS-ERG t(21;22)(q22;q12), EWS-ETV1 t(7;22)(p22;q12), EWS-E1AF t(17;22)(q12;q12), and EWSFEV t(2;22)(q33;q12) according to their frequency, respectively.^8^, ^9^ The detection of ESWR1-FLI1 rearrangement is not diagnostic for ES, also in rhabdomyosarcoma it has been observed.^10^ In this case, histopathologic-immunohistochemical evaluation in conjunction with molecular testing for ESWR1-FLI1 rearrangement provide a definitive diagnosis. While evaluating a patient for whom ES is suspected or diagnosed definitely, appropriate imaging methods should be performed as a first priority. Computed tomography is used to monitor bone destruction and mass propagation fields, while MRI is an ideal method for the evaluation of soft tissue involvement around the primary lesion.^9^ In our patient, we evaluated the tumor extension by MRI.

The aim of ES treatment is to maintain all normal functions and to prevent recurrences and sequellae in the long term while eliminating the disease. The recommended treatment is a combination of surgery with chemotherapy and radiotherapy. Early diagnosis followed by wide resection, chemotherapy, and radiotherapy can leave the patient disease-free for a long time. It has been reported that the recent use of proton beam therapy provides local control of the disease and prevents possible complications of radiotherapy on surrounding tissues (such as vision loss and intracranial complications).^9^, ^10^ In this case, after recurrences we added the radiotherapy to the chemotherapy treatment following the resurgery.

## Conclusion

Ewing's sarcoma is rare in the maxillary sinus. Surgery, chemotherapy, and radiotherapy should be used in combination to treat this lesion, which stems from high-grade tumors. Patients should be closely followed-up with due to the possibilities of local recurrence and distant metastasis.

## Conflicts of interest

The authors declare no conflicts of interest.
